# A Smooth Path between the Classical Realm and the Quantum Realm

**DOI:** 10.3390/e23121689

**Published:** 2021-12-16

**Authors:** John R. Klauder

**Affiliations:** Department of Physics and Department of Mathematics, University of Florida, Gainesville, FL 32611-8440, USA; klauder@ufl.edu

**Keywords:** affine quantization, field theory and gravity, unification of the classical and quantum realms

## Abstract

A simple example of classical physics may be defined as classical variables, *p* and *q*, and quantum physics may be defined as quantum operators, *P* and *Q*. The classical world of p&q, as it is currently understood, is truly disconnected from the quantum world, as it is currently understood. The process of quantization, for which there are several procedures, aims to promote a classical issue into a related quantum issue. In order to retain their physical connection, it becomes critical as to how to promote specific classical variables to associated specific quantum variables. This paper, which also serves as a review paper, leads the reader toward specific, but natural, procedures that promise to ensure that the classical and quantum choices are guaranteed a proper physical connection. Moreover, parallel procedures for fields, and even gravity, that connect classical and quantum physical regimes, will be introduced.

## 1. Introduction

This project is a review of several of the author’s articles, which feature several important consequences that apply to ‘Quantum Mechanics and Its Foundations’. After many decades, the issues that concern ‘quantization’ are still being debated within a variety of procedures. This paper proposes to offer a natural viewpoint in which the classical realm joins with the quantum realm in that we identify a ‘bridge’ that passes smoothly between these two realms, which have been normally treated as separate, disconnected, and distinct realms. We have drawn from published papers of the author, that may be examined, if needed, to have a deeper understanding of one or more topics.

We begin by establishing unique classical and quantum tools that preserve the physical role, i.e., beyond merely the mathematical role, of the necessary variables in each realm. This will include the traditional canonical quantization (CQ) tools, as well as spin quantization (SQ) tools (which are not considered much further), and as well as (relatively new) tools referred to as affine quantization (AQ) tools. These reliable tools are then used to examine various models, which run from harmonic oscillators, to field theory, to Einstein’s gravity, and well beyond.

For scalar fields, denoted by $\varphi_{n}^{p}$, where n=s+1 is the number of spacetime dimensions and *p* is the power of the interaction term, CQ can quantize those fields when p<2n/(n−2), but fails for those when p≥2n/(n−2), which involve nonrenormalizablity. Instead, AQ solves all such fields because it *eliminates nonrenormalizability!* Moreover, while CQ has difficulties with quantum gravity because the metric $g_{ab}\left(x\right)$ must be a positive matrix, AQ is *designed* to handle a positive metric!

While those topics are covered in this work, we need to start by introducing our tools.

### 1.1. A Brief Review of Canonical Quantization

Classical variables p&q that obey −∞<p&q<∞ and have a Poisson bracket {q.p}=1 are candidates to promote to basic quantum operators P&Q, which obey [Q,P]=iℏ11. For convenience, we choose q&Q as dimensionless, while p&P&ω (ω appears below) have the dimensions of *ℏ*. However, P&Q will be physically correct operators *provided* that the original variables p&q are ‘Cartesian coordinates’ [1].

#### Canonical Coherent States

Cartesian coordinates can be found in normalized coherent states of the form $|p,q\rangle \equiv e^{-iqP/\hbar }e^{ipQ/\hbar }\;|\omega \rangle$, with (Q+iP/ω)|ω〉=0, which implies that 〈ω|Q|ω〉=〈ω|P|ω〉=0. Although not mutually orthogonal, coherent states can formulate the identity, e.g.,

(1) $$\begin{array}{c} 1\;\;1=\int |p,q\rangle \langle p,q|\;do\;dq/2\pi \hbar \;, \end{array}$$

which will become an important relation later in the paper. For any operator expression, like H(P,Q), the coherent states lead to

(2) $$\begin{array}{c} \langle p,q|H(P,Q)|p,q\rangle =\langle \omega |H(P+p,Q+q)|\omega \rangle =H(p,q)+O(\hbar ;p,q)\;. \end{array}$$

The H(p,q) term is free of *ℏ*, which implies that the H(p,q)=H(p,q) as Dirac is also required [1]. Moreover, the whole line of (2) is *independent of any phase factor* of the coherent states, such as $|p,q:f\rangle =e^{if(p,q)}|p,q\rangle$. This independence is carried over to a Fubini–Study metric [2], which is deliberately designed to be independent of the phase *f*, and leads to

(3) $$\begin{array}{c} d\sigma {\left(p,q\right)}^{2}\equiv 2\hbar^{2}{\;[|\;|\;d|p,q\rangle |\;|}^{2}-{\left|\langle p,q|\;d|p,q\rangle \right|}^{2}]=\omega^{-1}\;dp^{2}+\omega \;dq^{2}\;. \end{array}$$

All that leads us to suitable Cartesian coordinates! More generally, this two-dimensional space may be called a ‘constant zero curvature’ surface. It is noteworthy that this ‘constant zero curvature’ was not *sought*, it was *created!* Efforts to use canonical quantization with classical variables that do not belong to a ‘constant zero curvature’ are very likely to lead to a physically incorrect quantization.

### 1.2. A Brief Review of Spin Quantization

The operators in this story are $S_{i}$ with i=1,2,3, and which (here $i=\sqrt{-1}\;$) satisfy $\left[S_{i},S_{j}\right]=i\;\hbar \;\epsilon_{ijk}\;S_{k}$. These operators obey $\Sigma_{l=1}^{3}\;S_{l}^{2}=\hbar^{2}s\left(s+1\right)1\;\;1_{2s+1}$, where 2s+1=2,3,4,… is the dimension of the spin matrices. The normalized eigenvectors of $S_{3}$ are $S_{3}|s,m\rangle =m\hbar |s,m\rangle$, where m∈{−s,…,s−1,s}.

#### Spin Coherent States

The spin coherent states are defined by

(4) $$\begin{array}{c} |\theta ,\varphi \rangle \equiv e^{-i\varphi S_{3}/\hbar }\;e^{-i\theta S_{2}/\hbar }\;|s,s\rangle \;, \end{array}$$

where −π<φ≤π, and −π/2≤θ≤π/2. It follows that

(5) $$\begin{array}{ccc} & & d\sigma {\left(\theta ,\varphi \right)}^{2}\\ & & \;\equiv 2\hbar^{2}{\;[\;|\;|\;d|\theta ,\varphi \rangle |\;|}^{2}-{\left|\langle \theta ,\varphi |\;d|\theta ,\varphi \rangle \right|}^{2}\;]=\left(s\hbar \right)\left[d\theta^{2}+\cos{\left(\theta \right)}^{2}\;d\varphi^{2}\;\right]\;. \end{array}$$

We can also introduce $q={\left(s\hbar \right)}^{1/2}\;\varphi$ and $p={\left(s\hbar \right)}^{1/2}\;\sin\left(\theta \right)$, along with |p,q〉=|p(θ,φ),q(θ,φ)〉, which leads to

(6) $$\begin{array}{ccc} & & \;d\sigma {\left(p,q\right)}^{2}\\ & & \;\equiv {2\hbar \;[\;|\;|\;d|p,q\rangle |\;|}^{2}-{\left|\langle p,q|\;d|p,q\rangle \right|}^{2}\;]={\left(1-p^{2}/s\hbar \right)}^{-1}dp^{2}+\left(1-p^{2}/s\hbar \right)\;dq^{2}\;. \end{array}$$

Equation (5) makes it clear that we are dealing with a spherical surface with a radius of ${\left(s\hbar \right)}^{1/2}$; this space is also known as a ‘constant positive curvature’ surface, and it has been created! These classical variables cannot lead to a physically correct canonical quantization. Instead, they offer a distinct quantization procedure that applies to different problems. However, Equation (6) makes it clear that if s→∞, in which case both *p* and *q* span the real line, we are led back to ‘Cartesian coordinates’, a basic property of canonical quantization (Using spin coherent states, is the resolution of the identity as given by $1\;\;1_{s}=\left(2s+1\right)\int |\theta ,\varphi \rangle \langle \theta ,\varphi |\;\cos\left(\theta \right)\;d\theta \;d\varphi /4\pi$).

This treatment of SQ will not be required in the following discussion. It was included because SQ is part of the family of constant curvatures of which SQ involves constant positive curvatures. This property will help us completely fill out the list of two-dimensional ‘constant curvatures’.

### 1.3. A Brief Review of Affine Quantization

Consider a classical system for which −∞<p<∞, but 0<q<∞, that does not lead to a self-adjoint quantum operator. Perhaps we can do better if we change classical variables. For example, the classical action factor pdq=pqdq/q=pqdln(q), leads to proper variables to promote to quantum operators. In particular, $d\equiv pq\to \left(P^{†}Q+QP\right)/2\equiv D\;\left(=D^{†}\right)$ (If Q>0, then $P^{†}\neq P$; however, $P^{†}Q=PQ$, so D=(PQ+QP)/2 as well). However, besides 0<q<∞, it may arise that −∞<q<0, or even −∞<q≠0<∞ (e.g., q≠0 may be helpful if $q^{-2}$ is part of a problem). To capture all three possibilities for *q*—and thus also for $Q\;(=Q^{†})$—we are led to [Q,D]=iℏQ. This symbol happens to be the Lie algebra of the “affine group” [3], and, incidentally, gives its name to affine quantization. Again, it is useful to choose dimensions such that q&Q are dimensionless while p&D have the dimensions of *ℏ*.

#### Affine Coherent States

The affine coherent states involve the quantum operators *D* and now Q>0, and we use the classical variables *p* and ln(q), with q>0. Specifically, we choose

(7) $$\begin{array}{c} |p;q\rangle \equiv e^{ipQ/\hbar }\;e^{-i\ln(q)\;D/\hbar }\;|\beta \rangle \;, \end{array}$$

where the fiducial vector |β〉 fulfills the condition [(Q−11)+iD/β]|β〉=0, which implies that 〈β|Q|β〉=1 and 〈β|D|β〉=0 (The semicolon in |p;q〉 distinguishes the affine ket from the canonical ket |p,q〉. All further uses of a semicolon signals that affine operators are involved in the construction of the relevant coherent states).

Affine coherent states also provide a resolution of identity, given, with b>ℏ/2, by

(8) $$\begin{array}{c} 1\;\;1=\int |p;q\rangle \langle p;q|\;dp\;dq/2\pi \hbar {\left[1-\hbar /2b\right]}^{-1}. \end{array}$$

Note: This equation will play a primary role in our program of the unification of classical and quantum realms for covariant scalar fields as well as for gravity.

Returning to the expectation of quantum operators using coherent states, we find that

(9) $$\begin{array}{c} \langle p;q|H^{\prime }\left(D,Q\right)|p;q\rangle =\langle \beta |H^{\prime }\left(D+pqQ,qQ\right)|\beta \rangle =H^{\prime }\left(pq,q\right)+O^{\prime }\left(\hbar ;p,q\right)\;, \end{array}$$

and, as ℏ→0, $H^{\prime }\left(pq,q\right)=H^{\prime }\left(pq,q\right)$, which is very much like what Dirac [1] required for CQ. It follows that the Fubini–Study metric, for q>0, becomes

(10) $$\begin{array}{ccc} & & d\sigma {\left(p;q\right)}^{2}\\ & & \;\equiv {2\hbar [|\;|\;d|p;q\rangle |\;|}^{2}-{\left|\langle p;q|\;d|p;q\rangle \right|}^{2}]=\beta^{-1}q^{2}\;dp^{2}+\beta \;q^{-2}\;dq^{2}\;. \end{array}$$

This expression leads to a surface that has a ‘constant negative curvature’ [4] of magnitude −2/β, which, like the other curvatures, has been ‘created’. This set of classical variables ca not lead to a physically correct canonical quantization. Instead, they offer a distinct quantization procedure that applies to different problems. Any use of classical variables that do not form a ‘constant negative curvature’ subject to an affine quantization is very likely to not be a physically correct quantization.

The rule that 0<q<∞ is limited, and we can easily consider 0<q+b<∞, where b>0. This changes the coherent states from ln(q) to ln(q+b), which then changes the Fubini–Study metric to $\beta^{-1}{\left(q+b\right)}^{2}\;dp^{2}+\beta \;{\left(q+b\right)}^{-2}\;dq^{2}$. If we choose to let b→∞ and at the same time let $\beta \to (\beta +\omega b^{2})$, we are led to $\omega^{-1}dp^{2}+\omega \;dq^{2}$, now with q∈IR, which, once again, applies to canonical quantization.

The three stories, about SQ, CQ, and AQ, complete our family of ‘constant curvature’ spaces. Additionally, the various coherent states can build ‘bridges’ in each case from the classical realm to the quantum realm, or also in the other way [5,6]. A simple example of a ‘bridge’, built with the help of coherent states, that connects classical and quantum realms, will be addressed in Section 1.6.

### 1.4. The Essence of Affine Quantization

Canonical quantization is the standard approach, but it can fail to yield an acceptable quantization, such as for a classical ‘half-harmonic oscillator’ with 0<q<∞. This very problem is easy to quantize with affine quantization; see [7,8]. Coherent states for affine quantization, with positive *q* and *Q* having passed their dimensions to *p* (or carried by *D*), rendering them dimensionless for simplicity, are given by

(11) $$\begin{array}{c} |p;q\rangle \equiv e^{ipQ/\hbar }\;e^{-i\ln(q)D/\hbar }\;|b\rangle \;, \end{array}$$

with [(Q−11)+iD/bℏ]|b〉=0. If $H^{\prime }\left(D,Q\right)$ denotes the quantum Hamiltonian, then a semiclassical expression called the ‘weak correspondence principle’ [9] is given by (Observe in this relation $H^{\prime }\left(pq,q\right)$ involves *ℏ* while $H^{\prime }\left(pq,q\right)$ does not involve *ℏ*).

(12) $$\begin{array}{ccc} & & H\left(p,q\right)\equiv H^{\prime }\left(pq,q\right)=\langle p;q|H^{\prime }\left(D,Q\right)|p;q\rangle \\ & & \;=\langle b|H^{\prime }\left(D+pqQ,qQ\right)|b\rangle \\ & & \;=H^{\prime }\left(pq,q\right)+O\left(\hbar ;p,q\right)\;, \end{array}$$

implying that when ℏ→0, leading to the standard classical limit, then $H^{\prime }\left(pq,q\right)=H^{\prime }\left(pq,q\right)$; namely, the quantum variables have the same functional positions as the appropriate classical variables. In addition, we find that these variables lead to a constant negative curvature surface (equal to −2/bℏ) as shown by the equation (Similar stories for canonical and spin quantizations appear in [10]).

(13) $$\begin{array}{c} d\sigma {\left(p,q\right)}^{2}\equiv \;2\hbar^{2}{\left[|\;|\;d|p;q\rangle |\;\right|}^{2}-{\left|\langle p;q|\;d|p;q\rangle \right|}^{2}{]=\left(b\hbar \right)}^{-1}q^{2}\;dp^{2}+\left(b\hbar \right)\;q^{-2}\;dq^{2}\;. \end{array}$$

This latter property, i.e., seeing that these particular classical variables arise from a constant negative curvature, renders them as favored coordinates, just like the favored variables of canonical quantization are those that are Cartesian coordinates, i.e., having a constant zero curvature [1].

After this background, we turn attention to the Schrödinger representation and equations for affine quantization. The quantum action functional (*q*), with normalized Hilbert space vectors, is given by

(14) $$\begin{array}{c} A_{q}=\int_{0}^{T}\langle \Psi \left(t\right)|\left[i\hbar \left(\partial /\partial t\right)-H^{\prime }\left(D,Q\right)\right]|\Psi \left(t\right)\rangle \;dt\;, \end{array}$$

and variational efforts lead to a form of Schrödinger’s equation

(15) $$\begin{array}{c} i\hbar \;(\partial |\Psi \left(t\right)\rangle /\partial t)=H^{\prime }\left(D,Q\right)\;|\Psi \left(t\right)\rangle \;. \end{array}$$

Schrödinger’s representation is Q→x and $D\to -i\frac{1}{2}\hbar \left[x\left(\partial /\partial x\right)+\left(\partial /\partial x\right)x\right]=-i\hbar \left[x\left(\partial /\partial x\right)+1/2\right]$, where 0<x<∞ (provided 0<Q<∞), and |Ψ(t)〉→ψ(x,t). This analysis leads to the familiar form of the Schrödinger equation

(16) $$\begin{array}{c} i\hbar \;\partial \;\psi \left(x,t\right)/\partial t=H^{\prime }\left(-i\hbar \left[x\left(\partial /\partial x\right)+1/2\right],x\right)\;\psi \left(x,t\right)\;. \end{array}$$

There is a new feature in affine quantization, one that is not in canonical quantization, namely that

(17) $$\begin{array}{c} Dx^{-1/2}=-i\hbar \left[x\left(\partial /\partial x\right)+1/2\right]\;x^{-1/2}=0\;. \end{array}$$

The analog of this relation in canonical quantization is P11=−iℏ(∂/∂x)11=0, which is self-evident, and leads to no useful relation.

#### 1.4.1. A Full-Harmonic Oscillator and CQ

The harmonic oscillator is a traditional example for CQ. We choose a Hamiltonian, $H=(p^{2}+q^{2})/2$, where −∞<p&q<∞, that involves only simple terms. From the CQ rules the quantum Hamiltonian is given by $H=(P^{2}+Q^{2})/2$. Using Schrödinger representation, wherein Q=x and leads to the equation for eigenvalues are derived from $\left(-\hbar^{2}\;\partial^{2}/\partial x^{2}+x^{2}\right)/2\;\psi_{n}\left(x\right)=E_{n}\;\psi_{n}\left(x\right)$. The eigenvalues are given by $E_{n}=\hbar \;\left(n+1/2\right)$, with n=0,1,2,3,…. The even n=0,2,4,… give even eigenvectors, $\psi_{2n}\left(-x\right)=\psi_{2n}\left(x\right)$, while the odd n=1,3,5,… eigenvalues lead to odd eigenvalues, $\psi_{2n+1}\left(-x\right)=-\psi_{2n+1}\left(x\right)$.

CQ has done well with the full-harmonic oscillator, but now we introduce the half-harmonic oscillator.

#### 1.4.2. A Half-Harmonic Oscillator and CQ

While the half-harmonic oscillator has the sane Hamiltonian, $H=(p^{2}+q^{2})/2$, we now require that 0<q<∞. There are two ways to examine this problem. If we choose to insist that Q>0, then $P^{†}\neq P$. This implies that there are (at least) two quantum Hamiltonians, $H_{0}=\left(PP^{†}+Q^{2}\right)/2$ and $H_{1}=\left(P^{†}P+Q^{2}\right)/2$. The energy levels are now $E_{0\;2n}=\hbar \left[\left(0,2,4,\ldots \right)+1/2\right]$, while $E_{1\;2n+1}=\hbar \left[\left(1,3,5,\ldots \right)+1/2\right]$. In the classical limit where ℏ→0, both quantum operators become $(p^{2}+q^{2})/2$. That implies that we can also mix the energy levels, e.g., E=ℏ[0,2,3,5,6,7,…)+1/2], signaling that there are infinitely many spectra, and this method fails (In fact, there are infinitely many self-adjoint Hamiltonian operators, e.g., $\{\left[P^{†\;r+2}/P^{r}+P^{r+2}/P^{†\;r}\right]/2+Q^{2}\}/2$ for all r=1,2,3,…, and they all have the same classical Hamiltonian, $(p^{2}+q^{2})/2$, with q>0).

A second way to examine this problem is to insist that −∞<q<∞, but add an infinite wall over x<0, which is so strong as to force the wave functions to zero when x<0. The usual operator in the region x>0 now leads to the usual eigenvectors for x>0. While the usual odd functions are the same for x>0, they pass continuously to zero for x<0. However, the usual even eigenvectors are discontinuous at x=0, and they cannot be accepted because any *P* (as in the Hamiltonian) acting on a discontinuous wave function does not lead to a normalizable result, and thus is not in the Hilbert space. Clearly, CQ cannot deal with a half-harmonic oscillator.

#### 1.4.3. A Half-Harmonic Oscillator and AQ

**Figure 1:**
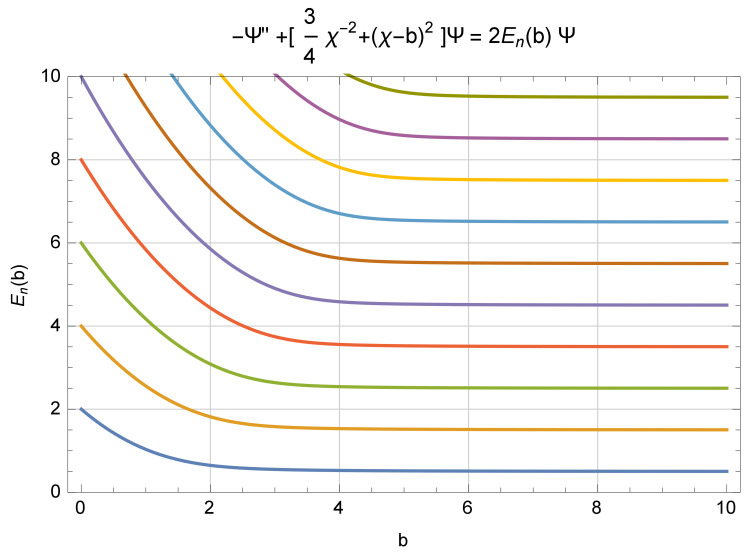
A graph of nine eigenvalues vs. *b*-value, showing gaps from 2ℏ toward 1ℏ. (The author of this figure has added a shift, x→x−b, for his version).

Since AQ is not designed to deal with a full-harmonic oscillator, we immediately admit that AQ fails on the full-harmonic oscillator, and we examine using AQ for a half-harmonic oscillator. Given that d=pq and q>0 are the classical affine variables, and $D=(P^{†}Q+QP)/2$ and Q>0, we are led to the affine Hamiltonian $H=(DQ^{-2}D+Q^{2})/2$, which then leads to the Schrödinger representation $H=[-\hbar^{2}\partial^{2}/\partial x^{2}+\left(3/4\right)\hbar^{2}/x^{2}+x^{2}]/2$. This equation [7,8] is known as a ‘spiked harmonic oscillator’, and its spectrum and eigenvectors have been established by L. Gouba. It is noteworthy that the spectrum is equally spaced, as that of the full-harmonic oscillator, but with a gap twice that of the full harmonic oscillator. There is an effort to move the end of the active space from x>0 to x>−b, for b>0. This changes the basic differential equation to become $H=[-\hbar^{2}\partial^{2}/\partial x^{2}+\left(3/4\right)\hbar^{2}/{\left(x+b\right)}^{2}+x^{2}]/2$. Figure 1 below, which was developed by C. Handy [11], shows clearly how the gap in the spectrum passes from 2ℏ for b=0, and toward 1ℏ, as *b* grows. As b→∞ the full set of eigenvectors and their spectrum are recreated when b=∞. That result is not available with CQ.

By now, the reader should recognize that AQ is a genuine expansion of CQ, and that AQ can truly solve certain quantum problems that CQ can not solve. Likewise, CQ can solve other quantum problems that AQ can not solve. An enlargement of valid quantization procedures offers a wider family of soluble problems. Interested readers are welcome to put AQ to work for themselves!

### 1.5. A Simple Example of the Unification of a Classical Realm and a Quantum Realm

In order to turn classical expressions into quantum expressions—specifically CQ expressions in this example—there are only three ingredients needed for any system [5]. For a simple, single classical example, with coordinates p&q, and with a Hamiltonian operator H(P,Q), Schrödinger’s equation is given by iℏ(∂|Ψ(t)〉/∂t)=H(P,Q)|Ψ(t)〉. Our next step is to introduce appropriate coherent states such as $|p,q\rangle =e^{-iqP/\hbar }\;e^{ipQ/\hbar }\;|\omega \rangle$, with 〈ω|ω〉=1 and 〈ω|Q|ω〉=〈ω|P|ω〉=0. These coherent state vectors span the appropriate Hilbert space with the identity operator 11=∫|p,q〉〈p,q|dpdq/2πℏ. Finally, we add the ‘bridge’

(18) $$\begin{array}{c} B\left(p,q|p^{\prime },q^{\prime }\right)\equiv \langle p,q|\left[i\hbar \left(\partial /\partial t\right)-H\left(P,Q\right)\right]|p^{\prime },q^{\prime }\rangle \;, \end{array}$$

which has been constructed from available parts. This tool then can be used to smoothly connect the classical and quantum realms. While the ‘bridge’ can easily lead to become the integrand for the classical action functional, namely

(19) $$\begin{array}{ccc} & & A_{c}=\int_{0}^{T}\langle p\left(t\right),q\left(t\right)|\left[i\hbar \left(\partial /\partial t\right)-H\left(P,Q\right)\right]|p(t,q\left(t\right)\rangle \;dt\\ & & \;=\int_{0}^{T}\langle \omega |\left[\left(p\left(t\right)+P\right)\dot{q}\left(t\right)-\dot{p}\left(t\right)Q-H\left(P+p\left(t\right),Q+q\left(t\right)\right)\right]|\omega \rangle \;dt\\ & & \;=\int_{0}^{T}\left\{p\left(t\right)\;\dot{q}\left(t\right)-H\left(p\left(t\right),q\left(t\right)\right)\right\}\;dt\;, \end{array}$$

where H(p,q)=H(p,q)+O(ℏ;p,q). The term O disappears if ℏ→0, but ℏ>0 in Nature. Normally, O is so tiny that it may be ignored, which then leaves behind the usual classical action functional. Indeed, we now know that beneath our glorious classical realm, as described by H(p,q), there is a tiny quantum story thanks to the fact that ℏ>0. Perhaps the more accurate statement is that 〈ω|H(P+p,Q+q)|ω〉=H(p,q)+O(ℏ;p,q), with 〈ω|P|ω〉=〈ω|Q|ω〉=0. The vector |ω〉 may vary depending on the local quantum background. Someday, we may be able to link our local |ω〉 vector with our local quantum background.

To set the stage, we take a longer way to achieve the classical realm with the classical action functional

(20) $$\begin{array}{ccc} & & \;A_{c}=\int_{0}^{T}\{\int \;\int \langle p\left(t\right),q\left(t\right)|p,q\rangle B\left(p,q|p^{\prime },q^{\prime }\right)\langle p^{\prime },q^{\prime }|p\left(t\right),q\left(t\right)\rangle \\ & & \;\times dp\;dq\;dp^{\prime }\;dq^{\prime }/{\left(2\pi \hbar \right)}^{2}\}\;dt\\ & & \;=\int_{0}^{T}\langle p\left(t\right),q\left(t\right)|\left[i\hbar \left(\partial /\partial t\right)-H\left(P,Q\right)\right]|p\left(t\right),q\left(t\right)\rangle )\;dt\;. \end{array}$$

For the quantum realm we can appeal to the resolutions of unity from the coherent states, along with the ‘bridge’; specifically

(21) $$\begin{array}{ccc} & & A_{q}=\int_{0}^{T}\{\int \;\int \;\langle \Psi \left(t\right)|p,q\rangle \langle p,q|B\left(p,q|p^{\prime },q^{\prime }\right)|p^{\prime },q^{\prime }\rangle \langle p^{\prime },q^{\prime }|\Psi \left(t\right)\rangle \\ & & \;\times dp\;dq\;dp^{\prime }\;dq^{\prime }/{\left(2\pi \hbar \right)}^{2}\}\;dt\\ & & \;=\int_{0}^{T}\langle \Psi \left(t\right)|\left[i\hbar \left(\partial /\partial t\right)-H\left(P,Q\right)\right]|\Psi \left(t\right)\rangle \;dt\;, \end{array}$$

which is the quantum action functional from which Schrödinger’s equation is derived. Similar examples hold true for both spin and affine coherent states and the ‘bridges’ they can create.

**Figure 2:**
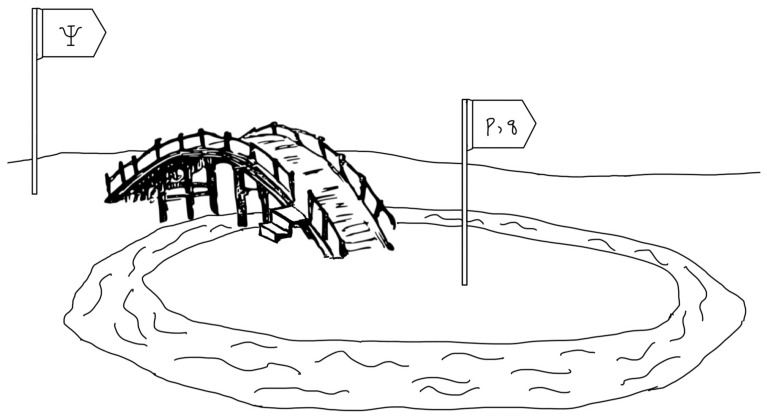
A representation of the ‘bridge’ connecting ‘quantum-land’ with ‘classical-land’. (This image was supplied by Jennifer Klauder and Dustin Wheeler).

Below, there is a cartoon showing the classical and quantum connected by a bridge; it is Figure 2.

### 1.6. Variations in How Wave Functions Appear in CQ and AQ

A conventional approach dealing with the operators P&Q is a Schrödinger representation in which Q→x, and −∞<x<∞. It follows that P→−iℏ∂/∂x. Conventional wave functions are ψ(x) and joining two wave functions leads to $\int \phi^{*}\left(x\right)\;\psi \left(x\right)\;dx$.

We reviewed this procedure for CQ because AQ has a different procedure.

#### Wave Functions for AQ

As expected, the principal quantum operators for AQ are D&Q. Again we choose Q→x, where now x>0 (or x<0, or just x≠0). We note that in CQ, the operator *P* acting on the unity operator, 11, leads to P11=0. As P1=0, then 1 acts as a very special wave function.

Since D1≠0, we ask if there is another pair of AQ operator-wave functions that lead to zero. At first, we find that $D\;\sum_{n=0}^{N}a_{n}Q^{n}\neq 0$, with $0\leq |a_{n}|<\infty$ and N<∞. In fact, $D\;Q^{-1/2}=0$, is the correct operator pair, and second factor, i.e., $x^{-1/2}$, can represent a particular form of wave function. Thus $x^{-1/2}$ can serve as a special wave function, such as, $\psi \left(x\right)=K\left(x\right)\;x^{-1/2}$. Support for this suggestion can be offered if we change Q→Q+b, which leads to $0=\frac{1}{\sqrt{1+b}}\;[\;\left[D+b\left(P^{†}+P\right)/2\right]\;{\left(Q+b\right)}^{-1/2}\to_{b\to \infty }P\;1\;\;1=0$, which recovers the special pair from CQ.

Our next two sections deal with covariant scalar fields and Einstein’s gravity. As the reader will observe, both topics can benefit from AQ.

## 2. The Unification of the Classical Realm and the Quantum Realm of Scalar Fields

### 2.1. Possible Results from Canonical Quantization

The conventional version of covariant scalar fields deals with the quantization of models given by the classical Hamiltonian

(22) $$\begin{array}{c} H_{c}\left(\pi ,\varphi \right)=\int \left\{\frac{1}{2}\left[\pi {\left(x\right)}^{2}+\left(\vec{\nabla }\varphi \right){\left(x\right)}^{2}+m_{0}^{2}\;\varphi {\left(x\right)}^{2}\;\right]+g_{0}\;\varphi {\left(x\right)}^{p}\;\right\}\;d^{s}\;x\;, \end{array}$$

where *p* is the (even, positive integer) power of the interaction term, *s* is the (positive integer) number of spatial dimensions (with n≡s+1 as the number of spacetime dimensions), $m_{0}^{2}>0$ is the mass term, and $g_{0}\geq 0$ is the coupling constant.

Canonical quantization leads to expected results for ‘free models’ (i.e., $g_{0}=0$) and all n≥2, while ‘non-free models’ (i.e., $g_{0}>0$) require that p<2n/(n−2). The case of p=4=n was determined to become “free’ by Monte Carlo and analytical studies [12,13,14], despite that $g_{0}>0$, and which probably, would also lead to ‘free’ results for the model with p=6 and n=3. The remaining models, where p>2n/(n−2), are nonreormalizable and, following a perturbation expansion of $g_{0}$, there is an infinite number of increasingly divergent terms; or, if treated as a whole, such models seem to collapse to ‘free theories’ with a vanishing interaction term despite the fact that $g_{0}>0$ [12,13,14].

Briefly summarized, CQ leads to unacceptable results whenever p>2n/(n−2). On the other hand, the classical analysis of cases where p>2n/(n−2) lead to natural and acceptable results.

We now show how models, for which p>2n/(n−2), can be successfully quantized using AQ rather than CQ.

### 2.2. Possible Results from Affine Quantization

The classical Hamiltonian in (22) is the same starting point, except that we replace the momentum field π(x) with the affine field κ(x)≡π(x)φ(x), with φ(x)≠0 because otherwise κ(x)=0 and π(x) can not help. This leads to the affine version of the classical Hamiltonian given by

(23) $$\begin{array}{c} H_{c}^{\prime }\left(\kappa ,\varphi \right)=\int \left\{\frac{1}{2}\left[\kappa^{2}\left(x\right)\varphi {\left(x\right)}^{-2}+\left(\vec{\nabla }\varphi \right){\left(x\right)}^{2}+m_{0}^{2}\;\varphi {\left(x\right)}^{2}\;\right]+g_{0}\;\varphi {\left(x\right)}^{p}\;\right\}\;d^{s}\;x\;, \end{array}$$

and the parameters *p*, *s*, $m_{0}^{2}>0$, and $g_{0}\geq 0$ have the same meaning as before.

The affine expression for the Hamiltonian requires that $0<\varphi {\left(x\right)}^{-2}<\infty$, which then implies that ${0<|\varphi \left(x\right)|}^{p}<\infty$, for all p<∞. *This fact forbids the appearance of any nonrenormalizable behavior*, which arise when a path in the Hamiltonian domain passes through φ(x)=0 with an inverse power that causes a divergent integration. As Monte Carlo studies show, $\varphi_{3}^{12}$ fails using CQ, but succeeds using AQ [15]. The same results also apply for the example $\varphi_{4}^{4}$.p [16] (The labeling of such models, $\varphi_{n}^{p}$, use n=s+1 as the number of space-time variables and *p* as the power of the interaction term).

Let us examine AQ for our scalar field. The basic affine operators are $\hat{\kappa }\left(x\right)\equiv \left[\hat{\pi }\left(x\right)\hat{\varphi }\left(x\right)+\hat{\varphi }\left(x\right)\hat{\pi }\left(x\right)\right]/2$ plus $\hat{\varphi }\left(x\right)\neq 0$, and they point toward the quantum commutator $\left[\hat{\varphi }\left(x\right),\hat{\kappa }\left(x^{\prime }\right)\right]=i\hbar \;\delta^{s}\left(x-x^{\prime }\right)\;\hat{\varphi }\left(x\right)$, again with $\hat{\varphi }\left(x\right)\neq 0$. The Schrödinger representation is $\hat{\varphi }\left(x\right)=\varphi \left(x\right)\neq 0$ and

(24) $$\begin{array}{c} \hat{\kappa }\left(x\right)=-i\frac{1}{2}\hbar \left[\varphi \left(x\right)\left(\delta /\delta \varphi \left(x\right)\right)+\left(\delta /\delta \varphi \left(x\right)\right)\varphi \left(x\right)\right]\;, \end{array}$$

which leads to an affine Schrödinger quantization of the quantum affine Hamiltonian given by

(25) $$\begin{array}{ccc} & & \;H^{\prime }\left(\hat{\kappa },\varphi \right)\\ & & =\int \left\{\frac{1}{2}\left[\hat{\kappa }\left(x\right)\varphi {\left(x\right)}^{-2}\hat{\kappa }\left(x\right)+\left(\vec{\nabla }\varphi \right){\left(x\right)}^{2}+m_{0}^{2}\;\varphi {\left(x\right)}^{2}\;\right]+g_{0}\;\varphi {\left(x\right)}^{p}\;\right\}\;d^{s}\;x\;. \end{array}$$

This appears to be only a ‘formal representation and equation’, since it is true that $\delta \varphi \left(x^{\prime }\right)/\delta \varphi \left(x\right)=\delta^{s}\left(x^{\prime }-x\right)$, and leads to *∞* if $x^{\prime }=x$.

To clarify the meaning of formal expressions requires some form of regularization. One example of a regularization is offered below. It is important to note that this particular topic involves more mathematics than physics, and for that reason we do not emphasize regularizations in this article.

The foregoing functional derivatives are derived from regularized procedures which replace φ(x) with a discrete basis that treats all of *x* as an *s*-dimensional lattice, $\varphi \left(x\right)\to \varphi_{k}$, and the usual space x→ka, $k\in {\left\{\cdots ,-1,0,1,2,3,\cdots \right\}}^{s}$, and a>0 is the tiny physical distance between rungs of the lattice. In this regularization,

(26) $$\begin{array}{c} {\hat{\kappa }}_{k}=-i\;\frac{1}{2}\;\hbar \;\left[\;\varphi_{k}\;\left(\partial /\partial \varphi_{k}\right)+\left(\partial /\partial \varphi_{k}\right)\;\varphi_{k}\;\right]\;\;a^{-s}\;. \end{array}$$

Additionally, $a^{s}$ is a tiny physical volume, and $ba^{s}$ (with b≃1) is a tiny dimensionless volume. This expression leads to ${\hat{\kappa }}_{k}\;\varphi_{k}^{-1/2}=0$, which, in the limit a→0, becomes $\hat{\kappa }\left(x\right)\;\varphi {\left(x\right)}^{-1/2}=0$.

Our example of a regularized Hamiltonian is given, with $J_{k,l}\equiv 1/\left(2s+1\right)$ for l=k and the 2s nearest spacial neighbors to k, by

(27) $$\begin{array}{ccc} & & \;H_{r}^{\prime }=\frac{1}{2}\;\sum_{k}\;{\hat{\kappa }}_{k}\;{\left(\Sigma_{l}J_{k,l}\;\varphi_{l}^{2}\right)}^{-1}{\hat{\kappa }}_{k}\;a^{s}+\frac{1}{2}\sum_{k,k^{*}}{\left(\varphi_{k^{*}}-\varphi_{k}\right)}^{2}\;a^{s-2}\\ & & \;+\frac{1}{2}\;m_{0}^{2}\;\sum_{k}\varphi_{k}^{2}\;a^{s}+g_{0}\;\sum_{k}\varphi_{k}^{p}\;a^{s}\;, \end{array}$$

where $k^{*}$ is one positive step forward from the site k for each of the *s* nearest spatial lattice sites, in which the site labels may be spatially periodic.

### 2.3. Affine Coherent States for Covariant Scalar Fields

In choosing suitable coherent states we need to deal with the fact that −∞<φ(x)≠0<∞ as well as $-\infty <\hat{\varphi }\left(x\right)\neq 0<\infty$. All coherent states include a fiducial vector [17], and in this case our normalized fiducial vector is chosen to obey the following relation,

(28) $$\begin{array}{c} \Pi_{x}\left[(\right|\hat{\varphi }\left(x\right)|-1\;\;1)+i\hat{\kappa }\left(x\right)/\beta \hbar ]\;|\beta \rangle =0\;, \end{array}$$

which ensures that $\langle \beta |\;|\hat{\varphi }\left(x\right)|\;|\beta \rangle =1$ and $\langle \beta |\hat{\kappa }\left(x\right)|\beta \rangle =0$. The chosen set of coherent states involve the basic AQ operators, and are given by

(29) $$\begin{array}{c} |\pi ;\varphi \rangle =e^{\left(i/\hbar \right)\int \pi \left(x\right)\;\hat{\varphi }\left(x\right)\;d^{s}\;x}\;e^{-\left(i/\hbar \right)\int \ln\left(|\varphi \left(x\right)|\right)\;\hat{\kappa }\left(x\right)\;d^{s}\;x}\;|\beta \rangle \;, \end{array}$$

and, drawing on this definition for our coherent states, the semi-classical Hamiltonian is given by

(30) $$\begin{array}{ccc} & & \;H^{\prime }\left(\kappa ,\varphi \right)=\langle \pi ;\varphi |H^{\prime }\left(\hat{\kappa },\hat{\varphi }\right)|\pi ;\varphi \rangle \\ & & \;=\langle \beta |H^{\prime }(\hat{\kappa }\left(x\right)+\pi \left(x\right)|\varphi \left(x\right)|\hat{\varphi }\left(x\right),|\varphi \left(x\right)|\hat{\varphi }\left(x\right))|\beta \rangle \\ & & \;=\langle \beta |H^{\prime }(\hat{\kappa }\left(x\right)+\pi \left(x\right)\varphi \left(x\right)|\hat{\varphi }\left(x\right)|,\varphi \left(x\right)|\hat{\varphi }\left(x\right)|)|\beta \rangle \\ & & \;=H^{\prime }\left(\kappa ,\varphi \right)+O\left(\hbar ;\pi ,\varphi \right)\;. \end{array}$$

For a suitable *L*, it follows that

(31) $$\begin{array}{ccc} & & \;d\sigma {\left(\pi ,\varphi \right)}^{2}=L\hbar^{2}{\left[\;|\;|\;d|\pi ;\varphi \rangle |\;\right|}^{2}-{\left|\langle \pi ;\varphi |\;d|\pi ;\varphi \rangle \right|}^{2}\;]\\ & & \;=\int \left\{{\left(\beta \hbar \right)}^{-1}\;\varphi {\left(x\right)}^{2}\;d\pi {\left(x\right)}^{2}+\left(\beta \hbar \right)\;\varphi {\left(x\right)}^{-2}\;d\varphi {\left(x\right)}^{2}\;\right\}\;d^{s}\;x\;. \end{array}$$

The result is a constant negative curvature, namely −2/βℏ, for each and every point *x*.

We next suggest expressions to act as potential solutions of Schrödinger’s equations. Specifically, we propose this regularized, and normalized, wave function

(32) $$\begin{array}{c} \Psi \left(\varphi \right)=\Pi_{k}W\left(\varphi_{k}\right){\left(ba^{s}\right)}^{1/2}\;\varphi_{k}^{-(1-2ba^{s})/2}\;, \end{array}$$

which fulfills its normalization

(33) $$\begin{array}{c} 1=\Pi_{k}\;\int \;|W\left(\varphi_{k}\right){|}^{2}\;\left(ba^{s}\right){\left|\varphi_{k}\right|}^{-(1-2ba^{s})}\;d\varphi_{k}\;. \end{array}$$

Finally, we introduce a Fourier transformation where

(34) $$\begin{array}{ccc} & & F\left(f\right)=\lim_{a\to 0}\;\Pi_{k}\int \;e^{if_{k}\varphi_{k}}\;{\left|W\left(\varphi_{k}\right)\right|}^{2}\;\left(ba^{s}\right)\varphi_{k}^{-(1-2ba^{s})}\;d\varphi_{k}\\ & & F\left(f\right)=\lim_{a\to 0}\Pi_{k}\{1-\left(ba^{s}\right)\;\int \left(1-e^{if_{k}\;\varphi_{k}}\right)|W\left(\varphi_{k}\right){|}^{2}d\varphi_{k}/\varphi_{k}^{\left(1-2ba^{s}\right)}\}\\ & & F\left(f\right)=\exp\{-b\int d^{s}\;x\;\int \left(1-e^{i\;f(x)\lambda }\right)\;|w\left(\lambda^{2}\right){|}^{2}d\lambda /|\lambda |\}\;, \end{array}$$

where W→w allows for changes in *W* when a→0.

### 2.4. The Unification of Classical and Quantum Covariant Scalar Fields

Drawing on Section 1.3 and Section 2.3, and their coherent states, we use |π;φ〉 to introduce a resolution of the identity,

(35) $$\begin{array}{c} 1\;\;1=N\int |\pi ;\varphi \rangle \langle \pi ;\varphi |\;D(\pi )\;D(\varphi ) \end{array}$$

for a suitable N, such as that provided by $1={N\int |\langle \beta |\pi ;\varphi \rangle |}^{2}\;D\left(\pi \right)\;D\left(\varphi \right)$.

Once again we introduce a ‘bridge’ for this exercise, namely

(36) $$\begin{array}{c} B\left(\pi ;\varphi |\pi^{\prime };\varphi^{\prime }\right)\equiv \langle \pi ;\varphi |\left[i\hbar \left(\partial /\partial t\right)-H\left(\hat{\pi },\hat{\varphi }\right)\right]|\pi^{\prime };\varphi^{\prime }\rangle \;, \end{array}$$

which is constructed from available ingredients.

For the classical action functional, we are led to the following procedure

(37) $$\begin{array}{ccc} & & \;A_{c}=N^{2}\;\int_{0}^{T}\{\int \;\int \langle \pi \left(t\right);\varphi \left(t\right)|\pi ;\varphi \rangle \;B\left(\pi ;\varphi |\pi^{\prime };\varphi^{\prime }\right)\langle \pi^{\prime };\varphi^{\prime }|\pi \left(t\right);\varphi \left(t\right)\rangle \;\\ & & \;\times \;D\left(\pi \right)\;D\left(\varphi \right)\;\;D\left(\pi^{\prime }\right)\;D\left(\varphi^{\prime }\right)\}\;dt\\ & & =\int_{0}^{T}\langle \pi \left(t\right);\varphi \left(t\right)|\left[i\hbar \left(\partial /\partial t\right)-H\left(\hat{\pi },\hat{\varphi }\right)\right]|\pi \left(t\right);\varphi \left(t\right)\rangle \;dt\\ & & =\int_{0}^{T}\left\{\pi \left(t\right)\;\dot{\varphi }\left(t\right)-H\left(\pi \left(t\right),\varphi \left(t\right)\right)\;\right\}\;dt\;, \end{array}$$

where H(π,φ)=H(π,φ)+O(ℏ;π,φ). If the term O(ℏ;π,φ) is so tiny it may be ignored, then H(π,φ)=H(π,φ), which becomes the usual classical Hamiltonian.

For the quantum action functional, we are led to the following procedure

(38) $$\begin{array}{ccc} & & \;A_{q}=N^{2}\;\int_{0}^{T}\{\int \;\int \langle \Phi \left(t\right)|\pi ;\varphi \rangle \;B\left(\pi ;\varphi |\pi^{\prime };\varphi^{\prime }\right)\langle \pi^{\prime };\varphi^{\prime }|\Phi \left(t\right)\rangle \;\\ & & \;\times \;D\left(\pi \right)\;D\left(\varphi \right)\;\;D\left(\pi^{\prime }\right)\;D\left(\varphi^{\prime }\right)\}\;dt\\ & & =\int_{0}^{T}\langle \Phi \left(t\right)|\left[i\hbar \left(\partial /\partial t\right)-H\left(\hat{\pi },\hat{\varphi }\right)\right]|\Phi \left(t\right)\rangle \;dt\;, \end{array}$$

which leads to Schrödinger’s equation when (38) is varied to expose its equations of motion.

Bravo ‘bridge’! And bravo AQ for ridding our analysis of nonrenormalizability!

## 3. The Unification of Classical Gravity and Quantum Gravity

### 3.1. Choosing the Right Classical Variables to Promote to Quantum Operators

For the gravity story, we seek the ingredients that will give us a gravity ‘bridge’. We focus on the classical Hamiltonian, which is the greatest difficulty in gravity quantization, especially if one uses canonical quantization. According to ADM [18], the traditional pair of variables are the momentum $\pi^{ab}\left(x\right)$ and the metric $g_{cd}\left(x\right)$, which is a strictly positive matrix, i.e., $ds{\left(x\right)}^{2}=g_{ab}\left(x\right)\;dx^{a}\;dx^{b}>0$, provided that $\{dx^{c}\}\neq 0$. The positive matrix of the metric implies that the classical momentum $\pi^{ab}\left(x\right)$ cannot become a self-adjoint quantum operator, and this is not acceptable when using canonical quantization. An affine quantization fares quite well with gravity, and our three ingredients will utilize the basic quantum operators of affine quantization. Notably, while the classical variables are the momentum $\pi^{ab}\left(x\right)$ and the metric $g_{ab}\left(x\right)$, where a,b,…=1,2,3, affine quantization dictates that we promote to operators the metric $g_{ab}\left(x\right)$ and the ‘momentric’ $\pi_{b}^{a}\left(x\right)\equiv \pi^{ac}\left(x\right)\;g_{bc}\left(x\right)$ (This symbol is called the ‘momentric’ field for its combination of *momen*tum and me*tric*). For affine quantization, therefore, we choose the basic quantum operators as ${\hat{g}}_{ab}\left(x\right)$ and ${\hat{\pi }}_{b}^{a}\left(x\right)$. Both of these operator fields can be self adjoint, and the metric operator ${\hat{g}}_{ab}\left(x\right)$ can be positive as desired.

### 3.2. Affine Quantization and Einstein’s Gravity

The standard Poisson bracket for the metric and momentum fields is given by

(39) $$\begin{array}{c} \left\{g_{ab}\left(x\right),\pi^{cd}\left(x^{\prime }\right)\right\}=\frac{1}{2}\;\delta^{3}\left(x,x^{\prime }\right)\left[\delta_{a}^{c}\delta_{b}^{d}+\delta_{a}^{d}\delta_{b}^{c}\right]\;, \end{array}$$

and the Poisson brackets for either two metric fields or two momentum fields would vanish. Instead, the set of Poisson brackets for the metric and momentric fields is given by

(40) $$\begin{array}{ccc} & & \left\{\pi_{b}^{a}\left(x\right),\pi_{d}^{c}\left(x^{\prime }\right)\right\}=\frac{1}{2}\;\delta^{3}\left(x,x^{\prime }\right)\;\left[\delta_{d}^{a}\;\pi_{b}^{c}\left(x\right)-\delta_{b}^{c}\;\pi_{d}^{a}\left(x\right)\;\right]\;,\\ & & \;\left\{g_{ab}\left(x\right),\;\pi_{d}^{c}\left(x^{\prime }\right)\right\}=\frac{1}{2}\;\delta^{3}\left(x,x^{\prime }\right)\;\left[\delta_{a}^{c}g_{bd}\left(x\right)+\delta_{b}^{c}g_{ad}\left(x\right)\;\right]\;,\\ & & \;\{g_{ab}\left(x\right),\;g_{cd}\left(x^{\prime }\right)\}=0\;. \end{array}$$

Observe that these Poisson brackets are true even if we change $g_{ab}\left(x\right)$ to $-g_{ab}\left(x\right)$, which permits us to restrict $\{g_{ab}\left(x\right)\}>0$. This is not possible with the Poisson bracket for the canonical variables.

#### 3.2.1. Affine Coherent States for Gravity

We choose the basic affine operators to build our coherent states for gravity [17]; specifcally,

(41) $$\begin{array}{c} |\pi ;\eta \rangle =e^{\left(i/\hbar \right)\int \pi^{ab}\left(x\right){\hat{g}}_{ab}\left(x\right)\;d^{3}\;x}\;e^{-\left(i/\hbar \right)\int \eta_{b}^{a}\left(x\right){\hat{\pi }}_{a}^{b}\left(x\right)\;d^{3}\;x}\;|\alpha \rangle \;\;\;\;[=|\pi ;g\rangle ]. \end{array}$$

The fiducial vector |α〉 has been chosen so that $\langle \alpha |{\hat{g}}_{ab}\left(x\right)|\alpha \rangle =\delta_{ab}$ as well as $\langle \alpha |{\hat{\pi }}_{a}^{b}\left(x\right)|\alpha \rangle =0$, and the matrix $\eta \left(x\right)\equiv \{\eta_{b}^{a}\left(x\right)\}$ enters the coherent states solely in the form given by

(42) $$\begin{array}{c} \langle \pi ;\eta |{\hat{g}}_{ab}\left(x\right)|\pi ;\eta \rangle ={\left[e^{\eta (x)/2}\right]}_{a}^{c}\;\langle \alpha |{\hat{g}}_{cd}\left(x\right)\;|\alpha \rangle \;{\left[e^{\eta (x)/2}\right]}_{b}^{d}={\left[e^{\eta (x)}\right]}_{ab}\equiv g_{ab}\left(x\right)\;, \end{array}$$

which preserves metric positivity, i.e., $\{g_{ab}\left(x\right)\}>0$. A companion relation is given by

(43) $$\begin{array}{c} \langle \pi ;\eta |{\hat{\pi }}_{b}^{a}\left(x\right)|\pi ;\eta \rangle =\pi^{ac}\left(x\right)\;g_{cb}\left(x\right)\equiv \pi_{b}^{a}\left(x\right)\;, \end{array}$$

which involves the metric result from (42). These relations allow us to rename the coherent states from |π;η〉 to |π;g〉.

As a consequence, the inner product of two gravity coherent states is given by

(44) $$\begin{array}{ccc} \langle \pi^{\prime \prime };g^{\prime \prime }|\pi^{\prime };g^{\prime }\rangle \; & & =\exp\{-2\int b\left(x\right)\;d^{3}x\\ & & \;\times \ln\left\{\frac{\det\{\frac{1}{2}\left[{g^{\prime \prime }}^{ab}\left(x\right)+{g^{\prime }}^{ab}\left(x\right)\right]+i\frac{1}{2\hbar }b{\left(x\right)}^{-1}\left[{\pi^{\prime \prime }}^{ab}\left(x\right)-{\pi^{\prime }}^{ab}\left(x\right)\right]\}}{\det{\left[{g^{\prime \prime }}^{ab}\left(x\right)\right]}^{1/2}\;\;\det{\left[{g^{\prime }}^{ab}\left(x\right)\right]}^{1/2}}\right\}\}\;. \end{array}$$

Here, the scalar density function b(x)>0 ensures the covariance of this expression.

To test whether or not we have ‘favored coordinates’ we examine, with a suitable factor *J*, the Fubini–Study metric given by

(45) $$\begin{array}{ccc} d\sigma {\left(\pi ,g\right)}^{2}\; & & \equiv J\hbar^{2}{[\;∥\;d|\pi ;g\rangle ∥}^{2}-{\left|\langle \pi ;g|\;d|\pi ;g\rangle \right|}^{2}\;]\\ & & =\int \{{\left(b\left(x\right)\hbar \right)}^{-1}g_{ab}\left(x\right)\;g_{cd}\left(x\right)\;d\pi^{bc}\left(x\right)\;d\pi^{da}\left(x\right)\\ & & \;+\left(b\left(x\right)\hbar \right)\;g^{ab}\left(x\right)\;g^{cd}\left(x\right)\;dg_{bc}\left(x\right)\;dg_{da}\left(x\right)\}\;d^{3}\;x\;. \end{array}$$

This metric, like those in earlier sections, represents a multiple family of constant negative curvature spaces. The product of coefficients of the differential terms is proportional to a constant rather like the previous affine metric stories. Based on the previous analysis we accept that the basic affine quantum variables have been promoted from basic affine classical variables.

The given choice of coherent states and their quantum operators therein have passed the test to involve constant negative curvature coordinates, which makes them favored affine coordinates for an affine quantization.

#### 3.2.2. Schrödinger’s Representation and Equation

Passing to operator commutation relations, the relations (42) and (43) point toward a promotion of the set of Poisson brackets to operator commutation relations given by

(46) $$\begin{array}{ccc} & & \left[{\hat{\pi }}_{b}^{a}\left(x\right),\;{\hat{\pi }}_{d}^{c}\left(x^{\prime }\right)\right]=i\;\frac{1}{2}\;\hbar \;\delta^{3}\left(x,x^{\prime }\right)\;\left[\delta_{d}^{a}\;{\hat{\pi }}_{b}^{c}\left(x\right)-\delta_{b}^{c}\;{\hat{\pi }}_{d}^{a}\left(x\right)\;\right]\;,\\ & & \;\left[{\hat{g}}_{ab}\left(x\right),\;{\hat{\pi }}_{d}^{c}\left(x^{\prime }\right)\right]=i\;\frac{1}{2}\;\hbar \;\delta^{3}\left(x,x^{\prime }\right)\;\left[\delta_{a}^{c}{\hat{g}}_{bd}\left(x\right)+\delta_{b}^{c}{\hat{g}}_{ad}\left(x\right)\;\right]\;,\\ & & \;[{\hat{g}}_{ab}\left(x\right),\;{\hat{g}}_{cd}\left(x^{\prime }\right)]=0\;. \end{array}$$

As with the Poisson brackets, these commutators are valid if we change ${\hat{g}}_{ab}\left(x\right)$ to $-{\hat{g}}_{ab}\left(x\right)$. For the metric and affine fields, we again find that we can choose the subset for which $\{{\hat{g}}_{ab}\left(x\right)\}>0$.

The classical Hamiltonian for our models is given [18] by

(47) $$\begin{array}{c} H^{\prime }\left(\pi ,g\right)=\int \left\{g{\left(x\right)}^{-1/2}\left[\pi_{b}^{a}\left(x\right)\pi_{a}^{b}\left(x\right)-\frac{1}{2}\pi_{a}^{a}\left(x\right)\pi_{b}^{b}\left(x\right)\right]+g{\left(x\right)}^{1/2}{\;}^{\left(3\right)}\;R\left(x\right)\right\}\;d^{3}x, \end{array}$$

where ${}^{\left(3\right)}\;R\left(x\right)$ is the three-dimensional Ricci scalar. For the quantum operators, we adopt a Schrödinger representation for the basic operators: specifically ${\hat{g}}_{ab}\left(x\right)=g_{ab}\left(x\right)$ and

(48) $$\begin{array}{c} {\hat{\pi }}_{b}^{a}\left(x\right)=-\frac{1}{2}i\hbar \;\left[\;g_{bc}\left(x\right)\;\left(\delta /\delta \;g_{ac}\left(x\right)\right)+\left(\delta /\delta \;g_{ac}\left(x\right)\right)\right)\;g_{bc}\left(x\right)\;]\;. \end{array}$$

Clarification of the last equation will be given when it is regularized. It follows that the Schrödinger equation is given by

(49) $$\begin{array}{ccc} & & i\hbar \;\partial \;\Psi \left(\left\{g\right\},t\right)/\partial t=\{\;\;\{\int \{\left[{\hat{\pi }}_{b}^{a}\left(x\right)\;g{\left(x\right)}^{-1/2}\;{\hat{\pi }}_{a}^{b}\left(x\right)-\frac{1}{2}{\hat{\pi }}_{a}^{a}\left(x\right)\;g{\left(x\right)}^{-1/2}\;{\hat{\pi }}_{b}^{b}\left(x\right)\right]\\ & & \;+g{\left(x\right)}^{1/2}{\;}^{\left(3\right)}\;R\left(x\right)\}\;d^{3}x\}\;\;\}\;\Psi \left(\left\{g\right\},t\right)\;, \end{array}$$

where {g} represents the $\left\{g_{ab}\left(\cdot \right)\right\}$ matrix field.

Much like the scalar field, we expect that the Schrödinger representation of eigenfunctions of the Hamiltonian operator have a ‘large field behavior’ and a ‘small field behavior’, and the Hamiltonian operator eigenfunctions are formally given by $\Psi \left(\left\{g\right\}\right)=W\left(\left\{g\right\}\right)\;\left[\Pi_{x}g{\left(x\right)}^{-1/2}\right]$, where the ‘small field behavior’ is formally obtained by the relation ${\hat{\pi }}_{b}^{a}\;F\left(g\right)=0$, which implies that $\left[g_{bc}\;\left(\partial /\partial g_{ac}\right)+\frac{1}{2}\delta_{b}^{a}\right]\;F\left(g\right)=0$ and it leads to $g_{bc}\;g^{ac}\;g\;\;dF\left(g\right)/dg+\frac{1}{2}\delta_{b}^{a}\;F\left(g\right)=0$. This requires that $g\;d\;F\left(g\right)/dg+\frac{1}{2}\;F\left(g\right)=0$, and hence $F\left(g\right)\propto g^{-1/2}$. In summary, we observe that

(50) $$\begin{array}{c} {\hat{\pi }}_{b}^{a}\left(x\right)\;g{\left(x\right)}^{-1/2}=0\;\;\;,\;{\hat{\pi }}_{b}^{a}\left(x\right)\;\Pi_{y}\;g{\left(y\right)}^{-1/2}=0\;. \end{array}$$

We next insert a brief, but relative, comment about the Hamiltonian operator constraints.

Using (50), the factor $g{\left(x\right)}^{-1/2}$ can be moved to the left in the Hamiltonian density; see (49). This permits changing the Hamiltonian density, essentially by multiplying the Hamiltonian density by $g{\left(x\right)}^{1/2}$, and using that expression to make the result a simpler approach to fulfill the Hamiltonian operator constraints [18] to seek Hilbert space states Ω({g}) such that

(51) $$\begin{array}{c} \left\{\left[{\hat{\pi }}_{b}^{a}\left(x\right)\;{\hat{\pi }}_{a}^{b}\left(x\right)-\frac{1}{2}{\hat{\pi }}_{a}^{a}\left(x\right)\;{\hat{\pi }}_{b}^{b}\left(x\right)\right]+g\left(x\right){\;}^{\left(3\right)}\;R\left(x\right)\right\}\;\Omega \left(\left\{g\right\}\right)=0\;. \end{array}$$

As were the earlier procedures, we regularize the chosen eigenfunctions by replacing the spacial continuum by a set of $N^{\prime }<\infty$ points labeled by the usual points ka and introduce a regularized (*r*) eigenfunction given by

(52) $$\begin{array}{c} \Psi_{r}\left(\left\{g\right\}\right)=W_{r}\left(\left\{g\right\}\right)\;\{\Pi_{k}\;{\left(ba^{3}\right)}^{1/2}\;{\left[\Sigma_{l}J_{k,l}\;g_{l}\right]}^{-1/2}\;, \end{array}$$

where the factors $J_{k,l}$ are the same factors as used earlier. Because the affine variable complex in (47) is not positive definite, the quantum eigenvalues will, most likely, range over the whole real line.

Thus, $W_{r}\left(\left\{g\right\}\right)$ will, again most likely, be positive and negative for all eigenfunctions, and we focus attention on an appropriate eigenfunction that is nonzero in the vicinity of very small values of *g*. Just as in the covariant scalar case, we choose the ‘large field behavior’ of the regularized quantum Hamiltonian operator from the classical Hamiltonian, and we choose the ‘small field behavior’ of the regularized quantum Hamiltonian, i.e., the term $\Pi_{k}{\left(ba^{3}\right)}^{1/2}{\left[\Sigma_{l}J_{k,l}\;g_{l}\right]}^{-(1-ba^{3})/2}$. We are led to the regularized form of the quantum Hamiltonian in the Schrödinger density representation given [10] by

(53) $$\begin{array}{c} H_{r}=\sum_{k}\left\{\;{\hat{\pi }}_{b\;k}^{a}\;\;J_{k}\left(g\right)\;{\hat{\pi }}_{a\;k}^{b}-\frac{1}{2}\;{\hat{\pi }}_{a\;k}^{a}\;\;J_{k}\left(g\right)\;{\hat{\pi }}_{b\;k}^{b}+g_{k}^{1/2}{\;}^{\left(3\right)}\;R_{k}\right\}\;a^{3}\;, \end{array}$$

where $J_{k}\left(g\right)\equiv {\left[\Sigma_{l}J_{k,l}\;g_{l}\right]}^{-1/2}$ and

(54) $$\begin{array}{ccc} & & {\hat{\pi }}_{b\;k}^{a}=-i\;\;\frac{1}{2}\;\hbar \left\{\frac{\partial }{\partial \;g_{ac\;k}}\;\;g_{bc\;k}+g_{bc\;k}\;\frac{\partial }{\partial \;g_{ac\;k}}\right\}\;a^{-3}\;. \end{array}$$

We have strongly focused on making the Hamiltonian operator well defined so that, when we consider the constraints, we are ensured that the operator will result in the correct properties.

### 3.3. The Unification of Classical and Quantum Gravity Realms

With that much background, we again choose three elements to effect our unification. Once again, we introduce Schrödinger’s equation $i\hbar (\partial \;|\Psi \left(t\right)\rangle /\partial t)=H^{\prime }\left({\hat{\pi }}_{b}^{a},{\hat{g}}_{cd}\right)\;|\Psi \left(t\right)\rangle$. Next, we recall our coherent states for gravity; in particular, in which $\eta {\left(x\right)}^{T}=\eta \left(x\right)$ and ${\left\{e^{\eta (x)}\right\}}_{ab}=g_{ab}\left(x\right)>0$, then

(55) $$\begin{array}{c} |\pi ;g\rangle =e^{-\left(i/\hbar \right)\int \pi^{ab}\left(x\right)\;{\hat{g}}_{ab}\left(x\right)\;d^{3}\;x}\;e^{\left(i/\hbar \right)\int \eta_{b}^{a}\left(x\right)\;{\hat{\pi }}_{a}^{b}\left(x\right)\;d^{3}\;x}\;|\alpha \rangle \;, \end{array}$$

where 〈α|α〉=1, $\langle \alpha |{\hat{g}}_{ab}\left(x\right)|\alpha \rangle =\delta_{ab}$, and $\langle \alpha |{\hat{\pi }}_{d}^{c}\left(x\right)|\alpha \rangle =0$. We observe that the gravity coherent states can also resolve their Hilbert space identity as $N^{\prime }\int \;|\pi ;g\rangle \langle \pi ;g|\;D\left(\pi \right)\;D\left(g\right)=1\;\;1$, where we use $N^{\prime }\int {\left|\langle \alpha |\pi ;g\rangle \right|}^{2}\;D\left(\pi \right)\;D\left(g\right)=1$ to help fix $N^{\prime }$.

We next introduce the gravity ‘bridge’ given by

(56) $$\begin{array}{c} B\left(\pi ;g|\pi^{\prime };g^{\prime }\right)\equiv \langle \pi ;g|\left[i\hbar \left(\partial /\partial t\right)-H^{\prime }\left({\hat{\pi }}_{b}^{a},{\hat{g}}_{cd}\right)\right]|\pi^{\prime };g^{\prime }\rangle \;. \end{array}$$

Once again, we assert that the ‘bridge’ is built from available material.

Like the earlier cases, the ‘bridge’ is created the classical action functional, specifically

(57) $$\begin{array}{ccc} & & A_{c}=N^{\prime 2}\int_{0}^{T}\{\int \;\int \langle \pi \left(t\right);g\left(t\right)|\pi ;g\rangle B\left(\pi ;g|\pi^{\prime };g^{\prime }\right)\langle \pi^{\prime };g^{\prime }|\pi \left(t\right);g\left(t\right)\rangle \\ & & \;\times D\left(\pi \right)\;D\left(g\right)\;\;D\left(\pi^{\prime }\right)\;D\left(g^{\prime }\right)\;\}\;dt\\ & & \;=\int_{0}^{T}\langle \pi \left(t\right);g\left(t\right)|[i\hbar \left(\partial /\partial t\right)-H\left({\hat{\pi }}_{b}^{a},{\hat{g}}_{cd}\right)|\pi \left(t\right);g\left(t\right)\rangle \;dt\\ & & \;=\int_{0}^{T}\left\{\int \left[\pi^{ab}\left(x,t\right)\;{\dot{g}}_{ab}\left(x,t\right)-H\left(\pi \left(x,t\right),g\left(x,t\right)\right)\right]\;d^{3}\;x\;\right\}\;dt\; \end{array}$$

where H(π,g)=H(π,g)+O(ℏ;π,q).

Our first use of the ‘bridge’ to create the quantum action functional, specifically

(58) $$\begin{array}{ccc} & & A_{c}=N^{\prime 2}\int_{0}^{T}\{\int \;\int \langle \Psi \left(t\right)|\pi ;g\rangle B\left(\pi ;g|\pi^{\prime };g^{\prime }\right)\langle \pi^{\prime };g^{\prime }|\Psi \left(t\right)\rangle \\ & & \;\times D\left(\pi \right)\;D\left(g\right)\;\;D\left(\pi^{\prime }\right)\;D\left(g^{\prime }\right)\;\}\;dt\\ & & \;=\int_{0}^{T}\langle \Psi \left(t\right)|[i\hbar \left(\partial /\partial t\right)-H\left({\hat{\pi }}_{b}^{a},{\hat{g}}_{cd}\right)|\Psi \left(t\right)\rangle \;dt\;, \end{array}$$

which leads to Schrödinger’s equations by varying the appropriate vectors.

Further suggestions on dealing with first and second class constraints in order to render the AQ approach to quantize gravity may be found in [10].

### 3.4. Summary

Our efforts have been to show that affine quantization is a worthy procedure to join canonical quantization, especially because affine quantization can solve problems that cannot be solved by canonical quantization. The importance of coherent states in this effort shows how they can easily point to favored classical variables that lead to physically correct basic quantum operators, and which also serve to help create a ‘bridge’ that unifies the classical and quantum realms. Several examples have illustrated how these features work together to help advance the solution of otherwise essentially unsolved problems.

## Data Availability

Not applicable.
